# Extraskeletal Ewing's sarcoma presenting as a mediastinal mass with vena cava superior syndrome: A rare case report

**DOI:** 10.1016/j.ijscr.2024.109724

**Published:** 2024-04-30

**Authors:** Lakcandra Amar Amori, Isnin Anang Marhana, Dwi Wahyu

**Affiliations:** Department of Pulmonology and Respiratory Medicine, Faculty of Medicine, Universitas Airlangga – RSUD Dr. Soetomo, Surabaya, Indonesia

**Keywords:** Extraskeletal Ewing's sarcoma, Malignant round cell, Case report

## Abstract

**Introduction:**

Extraskeletal Ewing's Sarcoma is a rare entity of sarcoma that develops rapidly within soft tissue in any anatomic region, and the symptoms depend on its location.

Case presentation: The X-ray examination of a 28-year-old man with shortness of breath, cough, weight loss, and chest pain showed malignant round cell, in which confirmed by immunohistochemical examination. The examination indicated positive Vimentin findings in the cytoplasm and positive FLI-1 in the nuclei of the tumour cells. The diagnosis was consistent with extraskeletal Ewing's sarcoma. The patient submitted to a chest conference and received radiotherapy related to SVCS before debulking surgery.

**Discussion:**

The diagnostic challenges associated with Ewing's sarcoma may arise due to its diverse histological spectrum. Further examination is required in order to distinguish Ewing's sarcoma from other tumours, as its radiological specificity is limited. A multimodal approach for treatment and therapy is necessary to highlight the specific requirements of the patient's condition.

**Conclusion:**

Imaging modalities including X-rays and thoracic CT scans, supported by histopathological examination and immunohistochemistry, are essential for accurately diagnosing Extraskeletal Ewing's sarcoma. A multimodal approach may be considered as the best treatment for the patient with mediastinal Ewing's sarcoma.

## Highlights

• Extraskeletal Ewing's Sarcoma (EES) is a rare tumor often presenting as a large soft-tissue mass that lacks specific radiologic characteristics.
• The CT scan revealed a solid mass with irregular edges and necrotic components in the anteromedius mediastinum suggesting a malignant tumor.
• The patient refused the pro-embolization radio and doxorubicin-ifosfamide chemotherapy, thereby Ibandronate was chosen for 6 cycles.
• A 3 months-follow up of the patients concluded that the prognosis for patients is dubia ad malam.

## 1 Introduction

Extraskeletal Ewing's Sarcoma (EES) is a tumour in the Ewing's Sarcoma (ES) family that considered rare. This condition is not associated with gender or race. It causes localised pain and develops in soft tissues, commonly in the upper thigh, buttocks, arms, and shoulders. The symptoms manifest based on the presence of metastases and their primary site, generally in the lungs, bones, and bone marrow [1].

ES is a very aggressive tumour that mostly affects young adults and adolescents and widely known to be originated from mesenchymal progenitor cells [2]. EES commonly manifests as a large soft-tissue mass in the paraspinal region or lower extremity, exhibiting a radiologic image that lacks specific characteristics [3]. The radiologic evaluation of ES aids in detecting, assessing, and monitoring the disease prior to treatment, during metastasis or recurrence, and throughout the therapy [4]. In this study, we report a case of a patient diagnosed with EES who underwent debulking surgery via a left anterolateral thoracotomy incision and was prescribed Ibandronate in accordance to surgical case report (SCARE) 2023 guideline [5].

## 2 Case presentation

**Fig. 1:**
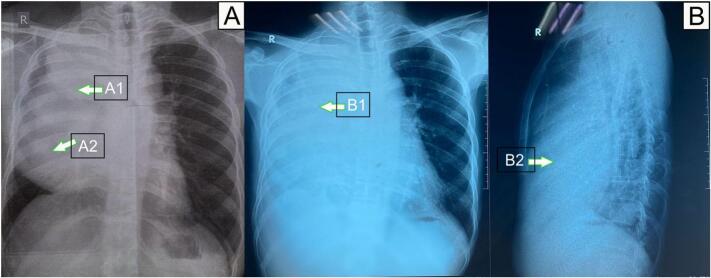
Chest photo: (A) AP shows homogeneous opacity of the right hemithorax with suspicion of a mass (A1), right pleural effusion (A2) and (B) PA/lateral dextra shows homogeneous opacity of the right hemithorax with suspicion of a mass (B1, B2).

A 28-year-old male patient was admitted to the Emergency Room (ER) exhibiting symptoms of shortness of breath, chest pain, and weight loss of approximately 4 kg in the past 3–4 months. The patient has no family history of cancer. The evaluation via X-ray imaging revealed a homogeneous opacity of the right hemithorax (Fig. 1).

**Fig. 2:**
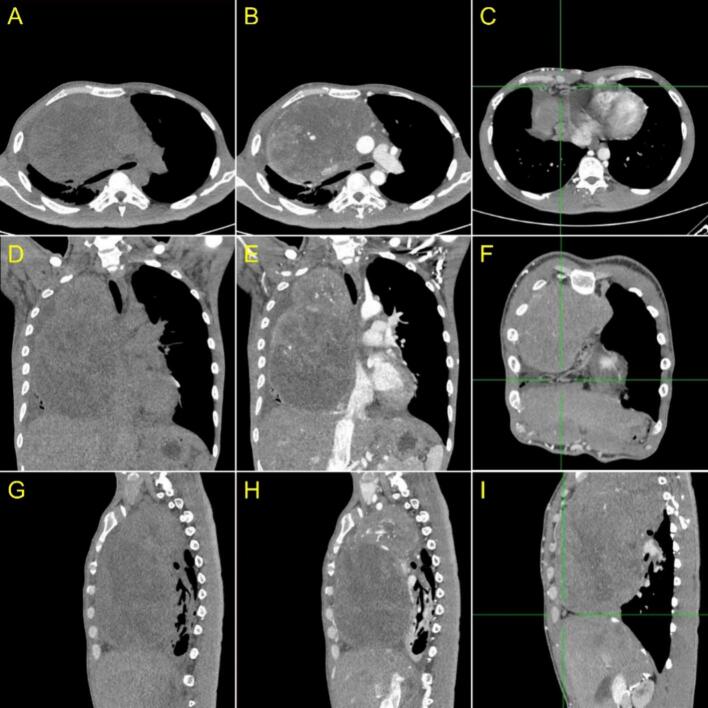
A CT Scan of the thorax. (A, D, G) Pre-contrast axial, sagittal, coronal CT scan thorax and (B, E, H) axial, sagittal, coronal CT Scan Thorax with contrast showed a solid mass with a necrotic component in the anteromedius mediastinum, which showed contrast enhancement. There was a mass enclosing the right pulmonary artery and vein, non-visualized superior vena cava with the impression of a tumour thrombus reaching the right atrium accompanied by (C, F, I) collateral veins (+) (green line area) in the mediastinum and right posterior thoracic wall with dilatation of the inferior vena cava.

The CT scan revealed a solid mass with irregular edges and necrotic components measuring approximately 12.1 × 16 × 20.7 cm in the anteromedius mediastinum. The mass exhibited contrast enhancement, indicating a malignant tumour that potentially causing superior vena cava syndrome (SVCS). Furthermore, a subcentimeter lymph node was identified near the left upper paratracheal area, as well as a calcified lump in the anterior segment of the left lung's superior lobe (Fig. 2).

**Fig. 3:**
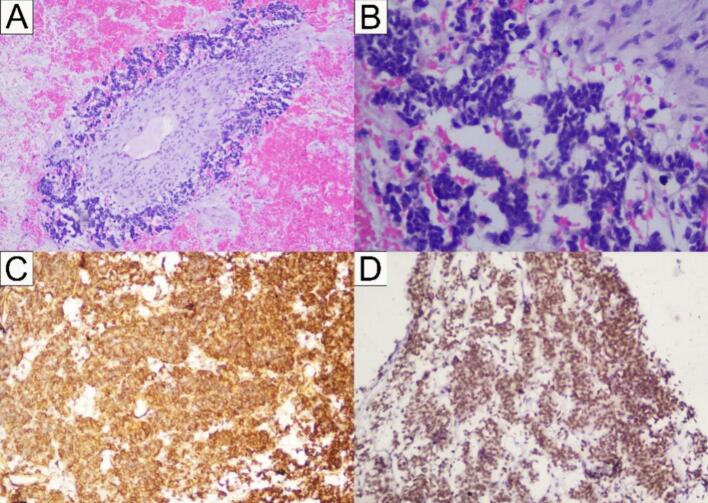
PA histology slides obtained (A and B) malignant round cells. On immunohistochemical examination, (C) Vimentin is positive in the cytoplasm of tumour cells, and (D) FLI-1 is positive in the nucleus of tumour cells, consistent with extraskeletal Ewing's sarcoma.

The outcomes of an ultrasound-guided fine needle aspiration biopsy (FNAB) examination revealed the presence of malignant round cells. The immunohistochemistry analysis demonstrated the cytoplasmic presence of Vimentin and the nuclear presence of FLI-1 in the tumour cells, indicating the presence of EES (Fig. 3).

**Fig. 4:**
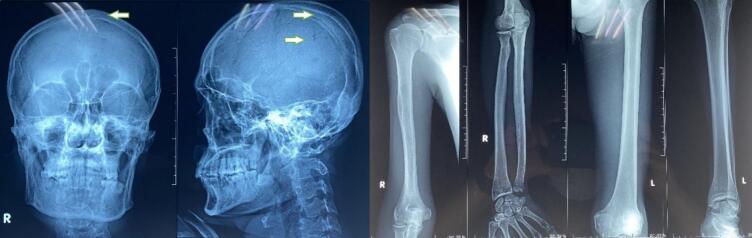
Bone survey examination, osteolisys in the os. calvaria (arrows).

Osteolysis in the Os. Calvaria was revealed in the bone survey as illustrated in Fig. 4, which may indicate a metastatic process.

**Fig. 5:**
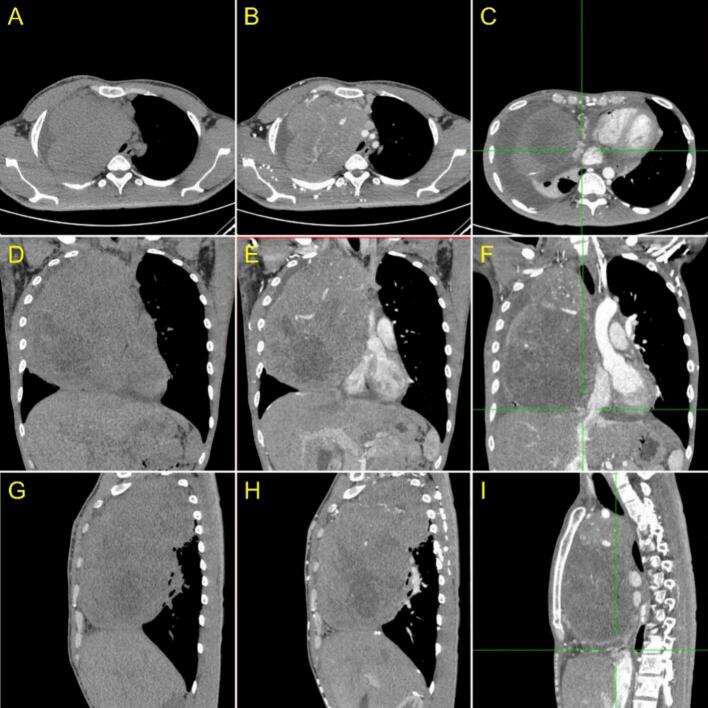
A CT-Scan of the thorax. (A, D, G) Pre-contrast axial, sagittal, coronal CT scan thorax and (B, E, H) axial, sagittal, coronal CT Scan Thorax with contrast shows a mediastinal mass with a solid component with a necrotic, the necrotic area is widespread, in the anteromedius mediastinum where contrast enhancement was visible when contrast administered. A mass appeared to encase the right pulmonary artery and vein, non-visualized superior vena cava, impression of tumour thrombus to the right atrium with (C, F, I) Collateral veins (+) (green line area) in the mediastinum and right posterior thoracic wall with dilatation of the inferior vena cava.

The CT scan revealed a malignant mediastinal mass with SVCS. Additionally, the recent findings represent the presence of bilateral pleural effusion, numerous left pulmonary nodules, ground glass opacity in the anterior basal lobe of the left lung, corpus osteolytic lesions VTh 3, 9, 12, and VL 1, as well as paraortic lymphadenopathy, subcentimeter in the subcarina and the peritumoural (Fig. 5).

**Fig. 6:**
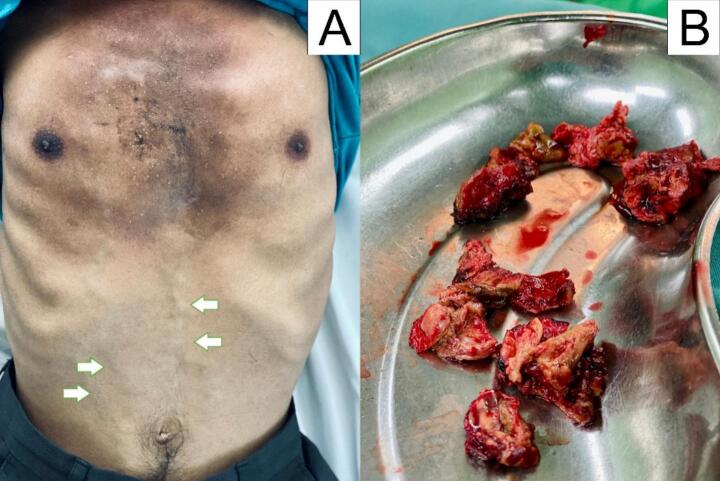
(A) Patient clinical shows collateral veins on the abdominal wall (arrows) (B) Tumour tissue obtained during tumour debulking.

A debulking surgery was conducted using a right anterolateral thoracotomy incision, which led to significant bleeding that gradually deepened layer by layer until it penetrated the pleura. The tumour is observed to completely occupy the right chest cavity, with adhesions present on multiple sides (Fig. 6).

Following a chest conference, the patient refused the pro-embolization radio intervention treatment and doxorubicin-ifosfamide chemotherapy, therefore Ibandronate was supplied to the patient for 6 cycles.

**Fig. 7:**
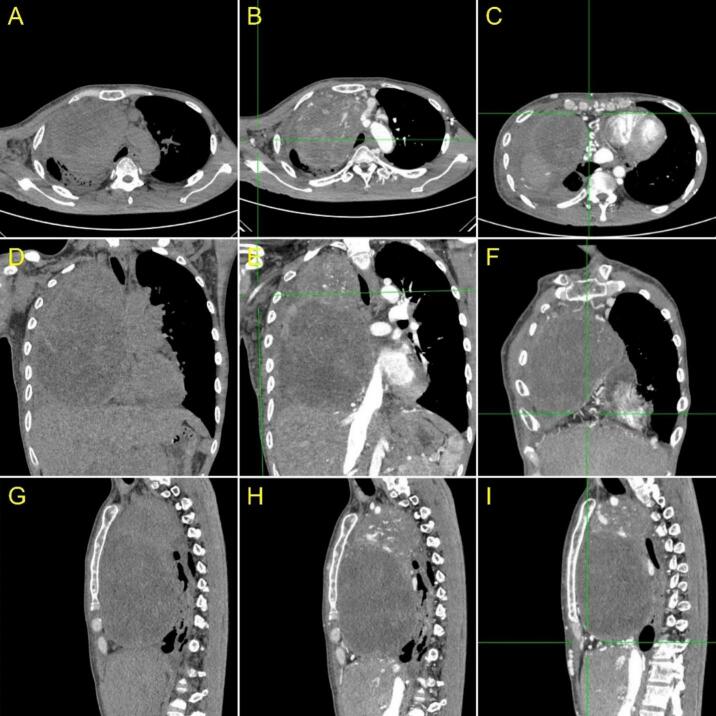
A CT-Scan of the thorax. (A, D, G) Pre-contrast axial, sagittal, coronal CT scan thorax and (B, E, H) axial, sagittal, coronal CT Scan Thorax with contrast showed a solid mass with a necrotic component in the anteromedioposterior mediastinum where contrast enhancement was visible, and (B,E) there was enlargement of right axilla (green line area). A mass appeared to encase the right pulmonary artery and vein, and the superior vena cava accompanied by (C, F, I) Collateral veins (+) (green line area).

A 3 months-follow up of the CT scan examination following the last surgery revealed an enhancing solid mediastinal mass measuring approximately 11.8 × 15.8 × 20.8 cm in the suspected SVCS. Multiple nodules in the left lung indicate the existence of metastases. Lymphadenopathy was observed in the suitable supraclavicular and axillary regions, along with bilateral pleural effusion. Subcentimeter lesions were found in the left subclavicle and upper paratracheal area. A lytic lesion was identified on the left pedicle of VTh 10 and VL 1. It can be concluded that the prognosis for patients is dubia ad malam (Fig. 7).

## 3 Discussion

ES is the second most prevalent type of malignant bone tumour, primarily affecting adolescents in their second decade of life. It belongs to a highly metastatic sarcoma class. The highest occurrence rate is observed between the ages of 10 and 15, with children under 10 accounting for about 30 % of cases and adults over 20 accounting for another 30 %. ES is more prevalent in males than females, with a ratio of 3:1. James Ewing classified various forms of sarcomas, including EES. These sarcomas, which develop from mesenchymal progenitor cells, have the potential to affect various soft tissues and bones within the body [2].

ES tumours are characterised by specific chromosomal translocations, the most prevalent of which is the t(11;22)(q24;q12) translocation. This leads to the formation of the EWS-FLI-1 fusion gene in 85 % of cases. Less frequent translocations, such as t(21;12)(22;12), lead to the development of EWS-FRG fusion genes in the remaining situations [6].

EES is a rare subtype of ES that originates from outside the skeleton. It shares genetic translocations and diagnostic features with Ewing's sarcoma bone (ESB). EES is more common in adults and accounts for about 25 % of all cases of Ewing's sarcoma family of tumours (ESFT) [7].

Primary mediastinal EES is a rare disease that requires specialized knowledge and resources for diagnosis and treatment. Patients should be referred to specialized centres that have expertise in rare diseases, as the initial symptoms are often atypical and progressive by involving surrounding tissues [8,9].

The diagnosis of ES requires a comprehensive evaluation of the patient's medical history and a thorough physical examination to identify the signs including the rapid growth of masses, pain, intermittent fever, and constitutional symptoms. Concurrently, lymphadenopathy or acute symptoms may serve as diagnostic indicators [7,10].

The patient exhibited symptoms of dyspnoea exacerbated by lying down, a cough without phlegm, chest pain aggravated by coughing, and a weight loss of 4 kg in the previous three months. Weight loss is a typical symptom that often occurs in malignancy, whereas chest pain and respiratory symptoms may be caused by a mass effect in the mediastinal cavity.

SVCS is a condition of blood flow obstruction caused by the compression of the superior vena cava by a mass located in the mediastinal cavity. This obstruction manifests clinically as swelling in the face or neck and the upper extremities, as well as the presence of dyspnoea, coughing, and dilated chest veins [11].

During the physical examination, the patient demonstrated elevated jugular venous pressure, ectatic veins on the chest wall, and collateral veins in the abdomen. These findings indicate that the venous congestion occurs due to the mass exerting pressure. Additional mass effects that were observed including an asymmetrical chest shape with limited left hemithorax movement, decreased vocal fremitus, dullness of percussion, and decreased breathing sounds in the right hemithorax.

A comprehensive clinical history and physical examination, as well as CT imaging are crucial in patients exhibiting symptoms of SVCS and a suspected malignancy in order to determine the urgency of medical intervention and establish a definitive diagnosis [12]. Imaging is an important modality for establishing a diagnosis, and it is also related to EES.

The imaging features commonly associated with EES include large heterogeneous soft tissue masses that frequently reflect tumour necrosis or haemorrhage, with rare displacement, calcification, and potential involvement of the bone surface, but without affecting the marrow cavity or showing normal fatty marrow attenuation [7,13].

The initial imaging modalities employed on this patient were lateral chest radiographs and anterior-posterior radiographs. The patient's chest x-ray showed an increasing homogeneous opacity in the right hemithorax indicating the presence of a potential mass, hence a CT scan of the chest with contrast was performed as a follow-up imaging procedure in response to the patient's chest x-ray.

CT imaging is the most preferred modality for diagnosing and evaluating EES due to its ability to offer detailed information on the tumour's size, enhancing patterns, the presence of necrosis, and potential invasion of surrounding structures On the other hand, MRI is commonly used to evaluate tumour invasion in the chest wall [7].

The MRI findings of EES typically demonstrate a moderate isointense signal on T1-weighted images, a moderately high signal on post-contrast T2-weighted images, and varying enhancement patterns. There is evidence of bleeding, necrosis, and occasional cystic degeneration. Additionally, invasion of surrounding tissue structures is commonly observed [8,13].

An increasing solid mass in the anteromedius mediastinum, pressing on the ascending aorta, heart, and trachea, was discovered by contrast chest CT scanning. It appeared to be malignant and caused SVCS. Additionally, subcentimeter left upper paratracheal and prevascular lymph nodes were observed, along with a calcified nodule in the anterior segment of the superior lobe of the left lung.

It is crucial to distinguish a mediastinal mass from other malignancies such as thymoma, lymphoma, teratoma, seminoma, endodermal sinus tumour, and solitary fibroma. Malignant thymoma with irregular mass in the anterior mediastinum resembles mediastinal ES, however it is more aggressive and lacking in both lymph node metastases and pleural effusion, and is frequently associated to myasthenia gravis. Histopathology and immunohistochemistry are necessary for accurate diagnosis due to limited radiological specificity [9].

A study suggests that non-calcified nodules >10 mm are more likely to be metastatic, while nodules <5 mm are more likely to be benign in bone and soft tissue sarcoma patients. Unfortunately, guidelines for managing these nodules remains limited [14].

ES has a diverse histological spectrum. The majority of cases (±80 %) exhibit a classic pattern with diffuse proliferation of small round cells. Uncommon forms of ES are including pattern neuroectodermal tumour (PNET) with Homer Wright-type rosetting, alveolar growth pattern resembling alveolar rhabdomyosarcoma, large cell pattern with prominent nucleoli, spindle cell pattern forming a faint rotating fascicle, nested epithelioid pattern with cohesive nests on a sclerotic background, and adamantinoma-like variants with basaloid squamous morphology. These patterns have distinctive features that may pose challenges in the diagnostic process [15].

The accurate diagnosis of ES relies on histological, immunohistochemical, and genetic studies. The presence of the t(11;22)(q24;q12) translocation, as well as the expression of CD99, FLI-1, and vimentin are commonly observed, which very helpful in identifying ES from other tumours [8].

The histopathological sample analysed in the present case report revealed a malignant round cell tumour that was classified under a differential diagnosis. The immunohistochemical analysis confirmed the presence of EES, as indicated by the expression levels of Vimentin and FL-1.

Currently, studies on the optimal treatments and prognostic factors for EES are still lacking. The current guidelines prescribe a combination of local treatment and chemotherapy, although uncertainties remain. Wide surgical resection has shown improved survival rates in EES compared to extraskeletal ESB [1].

In preclinical studies, CAR-T cells showed improved trafficking and infiltration into tumours with AMG102, suggesting intravenous administration as a suitable route to target EWS tumours with T cells [16].

Complete excision through surgery is essential for the treatment of local EES. Merely relying on surgery is insufficient, therefore it is recommended to employ a combination of surgery chemotherapy, and radiotherapy based on the tumour characteristics. Resection margins play a crucial role in ensuring local control. Chemotherapy is performed to extend progression-free survival in metastatic disease; nonetheless, the prognosis remains poor [1,17].

Multimodal treatment has improved the prognosis for ES, resulting in event-free survival rates of 65–72 % for localised disease and 21–28 % for metastatic disease. Second-line palliative chemotherapy using various regimens is the standard treatment during relapses [18].

Multimodality treatment incorporating chemotherapy significantly raises the 5-year survival rate for ES to 60–70 %. Systemic chemotherapy has notably improved survival by eliminating micrometastasis. The current regimen involves alternating cycles of specific drugs. However, chemotherapy alone is insufficient as a monotherapy option without surgery and radiotherapy [1].

Immunotherapy for ES remains challenging due to several factors including limited surface antigens, an immunosuppressive tumour microenvironment, and the absence of MHC class I leukocyte antigen molecules. The development of immunotherapy is currently on investigations including T-cell therapy, gene modification, surface target exploration, and combination approaches. These attempts aim to improve ES immunotherapy as well as serve as a guideline for proper future treatment [1].

In a study by Caltavituro et al. [9] en bloc resection was performed on mediastinal EES patients. The study demonstrated that the patients had no complications, and despite experienced relapse, the patients recovered from the disease completely. Patients involved in the study were candidates for adjuvant chemotherapy with vincristine, doxorubicin, and cyclophosphamide (VDC) alternating with ifosfamide and etoposide (IE).

Upon diagnosing SVCS, initial management involves elevating the patient's head. Treatment depends on the underlying cause, especially in malignancy cases. Appropriate chemotherapy or radiation therapy is recommended by multidisciplinary planning. A study suggested that SVCS patients with mediastinal tumours received pain medication, corticotherapy, and tumour-specific chemotherapy, resulting in rapid symptoms reduction and clinical improvement [12].

The patient in this study presented SVCS, thereby debulking surgery was performed following the radiotherapy. During surgery, a tumour rupture occurred, causing significant bleeding. Subsequently, a chest conference discussed the best management options, including radio intervention and chemotherapy, however the patient chose ibandronate administration instead. Education on the decision was provided at the POSA Polyclinic.

Ibandronic acid, a member of bisphosphonates group, is effective in preventing fractures, hypercalcemia, and osteoporosis. Bisphosphonates is a group of drugs used for osteoporosis and bone metastases, decrease cancer cell survival through HER receptor tyrosine kinases. They attach to bone minerals, inhibiting bone resorption and disrupting cell metabolism. Some bisphosphonates, like ibandronate, suggest potential benefits in treating bone malignancies and preventing complications [19].

Intravenous ibandronate (6 mg) or oral drug (50 mg) decreased the risk of skeletal-related events compared to placebo (risk ratio (RR) 0.80, 95 % CI 0.71 to 0.90, *p* = 0.002). The drug also capable to reduce the bone pain score significantly below the baseline compared to placebo at 96 weeks (weighted mean difference − 0.41, 95 % CI −0.56 to 0.27, *p* < 0.001). The incidence of diarrhoea, nausea and adverse renal events was similar between the ibandronate and placebo groups, however greater risk of abdominal pain was associated to ibandronate. The risk of skeletal-related events by ibandronate was comparable to another bisphosphonate drug, zoledronate (RR 1.02, 95 % CI 0.82 to 1.26, *p* = 0.87). The incidence of nausea, jaw osteonecrosis and fatigue was similar for the two drugs, however the incidence of adverse renal events was significantly lower in the ibandronate group. Overall, ibandronate significantly reduces the incidence of skeletal-related events and bone pain [20].

The pathogenesis of bone sarcomas is associated to the local bone remodeling dysregulation; and bisphosphonates, particularly nitrogen-bisphosphonates (N-BPs), have shown a potential in inhibiting bone resorption and tumour progression in preclinical studies. However, clinical trials have yet to demonstrate significant improvements, possibly due to the impact of N-BPs on macrophage differentiation and CD8 + -T lymphocyte infiltration. BP is an excellent platform for drug delivery to malignant bone sites with reduced systemic toxicity, thereby opening new opportunities for the future use [21].

The prognosis of EES is better compared with the skeletal subtype, although factors influencing prognosis appear to be similar in both subtypes. Notably, the overall 5-year survival rate was superior for local EES compared with local bone ES [1].

Worse prognosis in EES is associated with older age, pelvic involvement, high WBC, elevated LDH, low hemoglobin, and initial tumour size. Histological response to neoadjuvant chemotherapy is a decisive prognostic factor. Metastatic disease has a poor prognosis, while extremity lesions and surgically amenable lesions have a better prognosis. In mediastinal ES, large tumours, non-surgical treatment, and regional lymph node metastases are adverse prognostic factors [1,8].

The diagnosis for patient with EES should involves of a comprehensive evaluation on patient's medical history, physical examination, and imaging studies. Management for EES consists of a combination of surgery, chemotherapy, and radiotherapy, with wide surgical resection showing improved survival rates. However, the prognosis may be influenced by several factors such as age, tumour characteristics, and body response to treatment. In this case, a repeat CT scan showed the presence of an enhancing solid mass lung nodules suggestive of metastasis, lymphadenopathy, pleural effusion, and lytic lesions, indicating a poor prognosis for the patient.

## 4 Conclusion

Imaging examination including X-ray and chest CT scans, supported by histopathological examination and immunohistochemistry, are essential in establishing accurate diagnosis for EES.

The best treatment for the patient with mediastinal Ewing's sarcoma may involve a multimodal approach, including surgery, chemotherapy, and radiotherapy. Bisphosphonate therapy with ibandronate may also be considered in treating bone malignancies and preventing complications. However, the presence of metastasis and other factors indicate a poor prognosis, requiring aggressive treatment and close monitoring.

## Consent

Written informed consent was obtained from the patient to publish this case report and accompanying images. A copy of the written consent is available for review by the Editor-in-Chief of this journal on request.

## Ethical approval

Ethical approval was not required for this case report, however written informed consent was obtained from the patient and is available for review under request.

## Sources of funding

This research received no external funding.

## Authorship contribution statement

LAA, IAM, DW conceptualized and investigated the case; LAA drafted the manuscript and curated the data; IAM, DW supervising the manuscript; LAA searched the literature data; IAM, DW reviewed and revised the manuscript.

## Conflict of Interest

The authors declare the following financial interests/personal relationships which may be considered as potential competing interests: Isnin Anang Marhana reports a relationship with RSUD Dr Soetomo that includes: employment. Dwi Wahyu reports a relationship with RSUD Dr Soetomo that includes: employment. If there are other authors, they declare that they have no known competing financial interests or personal relationships that could have appeared to influence the work reported in this paper.

## Guarantor

Guarantor Isnin Anang Marhana is the person in charge of publishing our manuscript.

## Research registration

Not applicable.
